# Malakoplakia Masquerading As Urothelial Carcinoma: A Case Report and Review

**DOI:** 10.7759/cureus.84915

**Published:** 2025-05-27

**Authors:** Abdalla Aboelkheir, Bahaaeldeen Hesham, Senthilkumar Subramanian, Elham Mahjoor, Amgad Farouk, Hassan Hotait, Fariborz Bagheri

**Affiliations:** 1 Department of Urology, Dubai Hospital, Dubai, ARE; 2 Department of Clinical Sciences, College of Medicine, University of Sharjah, Sharjah, ARE; 3 Department of Family Medicine, Medcare Hospital, Dubai, ARE; 4 Department of Radiology, Medcare Hospital, Dubai, ARE; 5 Department of Pathology, Dubai Hospital, Dubai, ARE

**Keywords:** bladder mass, malakoplakia, michaelis-gutmann bodies, transurethral resection of bladder tumor, turbt, urothelial carcinoma mimicker

## Abstract

Malakoplakia is a rare inflammatory disorder primarily affecting the urinary tract, characterized by granulomatous reactions and Michaelis-Gutmann bodies. It typically presents with nonspecific urinary symptoms, mimicking malignancies radiologically and histologically, thereby complicating diagnosis. This case describes an uncommon presentation in an 18-year-old male with hematuria and dysuria, initially misdiagnosed as a urothelial neoplasm. Accurate diagnosis required extensive histopathological analysis, revealing classic malakoplakia features. Management included endoscopic resection and ureteral stenting. This emphasizes the diagnostic challenges posed by malakoplakia, the importance of careful multidisciplinary evaluation, detailed histopathology for correct identification, and the need for early intervention and follow-up to avoid renal complications.

## Introduction

Malakoplakia is a rare chronic inflammatory condition characterized histologically by histiocytes containing distinctive Michaelis-Gutmann bodies, intracellular calcified inclusions pathognomonic for this disorder [1,2]. Although it predominantly affects the genitourinary tract, occurrences in the gastrointestinal tract, skin, lungs, thyroid, salivary glands, and prostate have been documented, highlighting its broad anatomical spectrum [3-6]. Typically observed in middle-aged adults, malakoplakia displays a marked female predominance, especially in cases involving the urinary tract [7,8]. Clinically, the condition manifests through nonspecific symptoms such as fever, dysuria, hematuria, and pain, complicating diagnosis and clinical management due to its mimicry of malignant pathologies both clinically and radiologically [9,10].

## Case presentation

An 18-year-old male patient was referred to the urology clinic, initially presenting with terminal dysuria and mild gross hematuria. He was initially managed with Cefixime (400 mg/day) but later developed a fever (>38°C), prompting an ER visit. Lab results showed a creatinine level of 0.9 mg/dL, CRP elevated at 36 mg/L, and WBC at 3.7 × 10³/µL. Urinalysis was unremarkable, and urine culture showed no bacterial growth.

**Figure 1:**
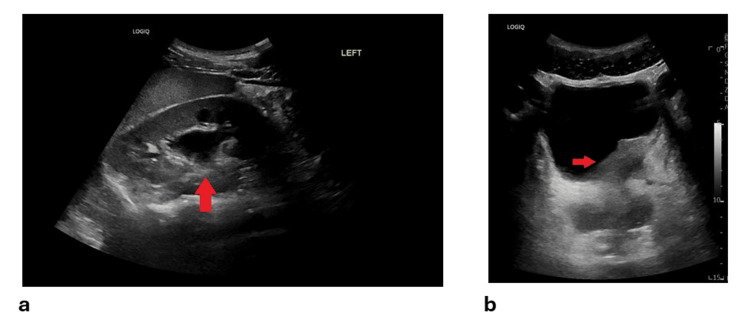
Ultrasonic images of the urinary tract. Ultrasound images showing (a) an arrow pointing to hydronephrosis of the left kidney, and (b) an arrow pointing to focal bladder wall thickening with a hypoechoic lesion around the left vesicoureteric junction.

**Figure 2:**
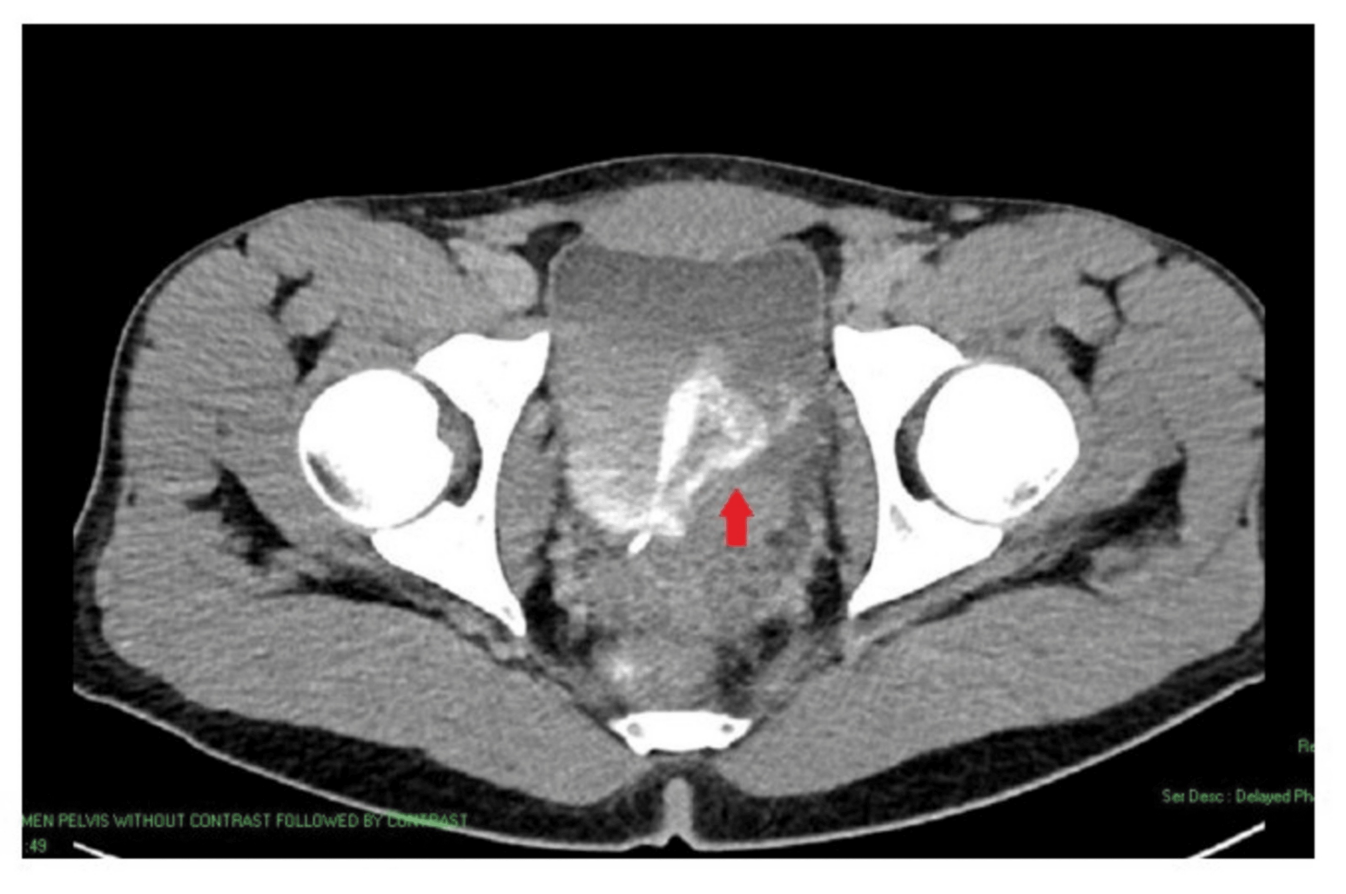
An axial cut of a CT scan taken in the contrast delayed phase. An arrow points to thickening of the left posterolateral aspect of the bladder wall, measuring approximately 4.5 × 4.3 × 3 cm, with internal nodularities observed.

**Figure 3:**
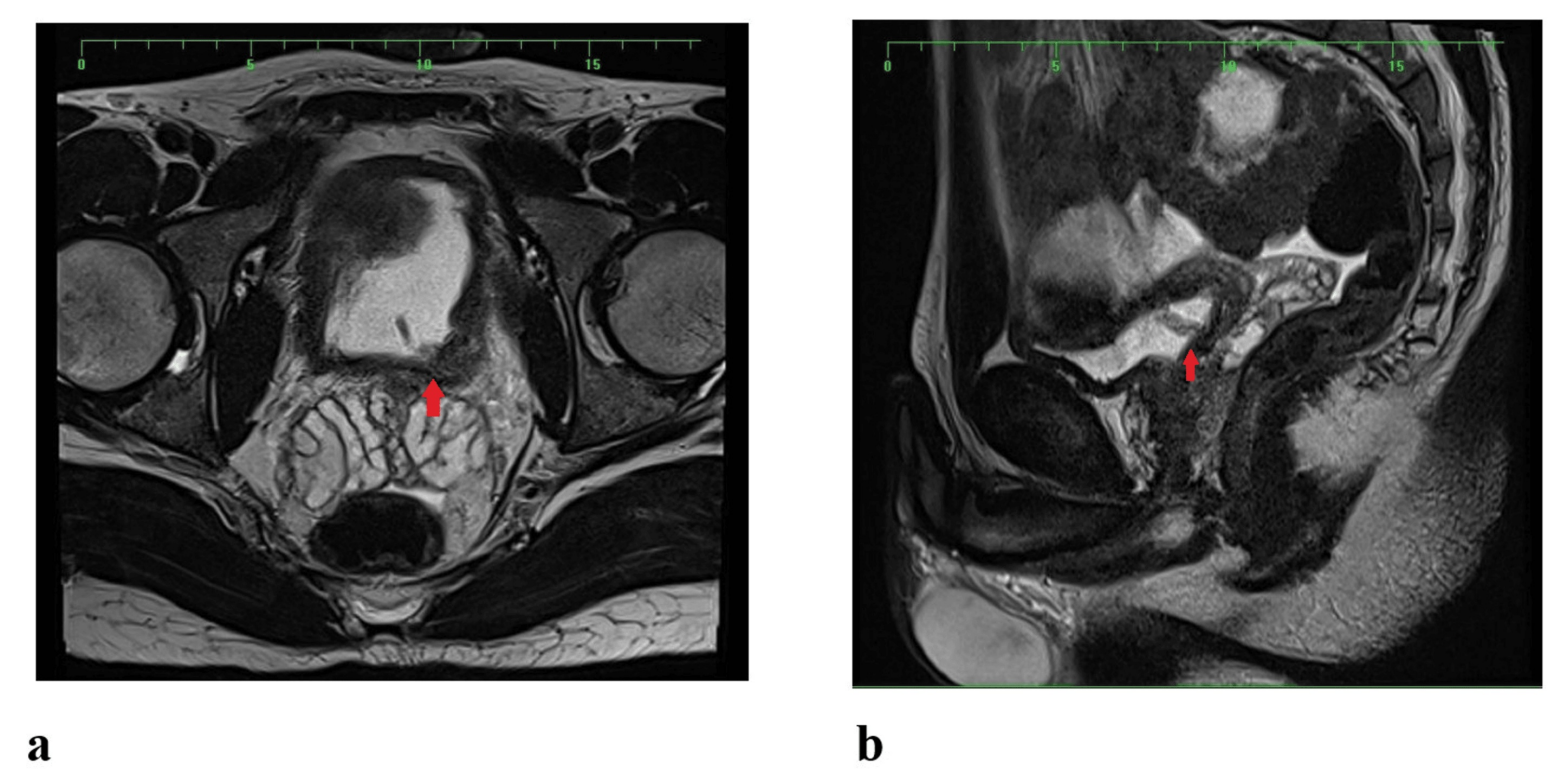
MRI showing possible extravesical involvement and loss of surrounding fat planes. Images taken in the (a) axial and (b) sagittal views, with arrows pointing to an enhancing, partially exophytic mass showing possible extravesical involvement and loss of surrounding fat planes.

**Figure 4:**
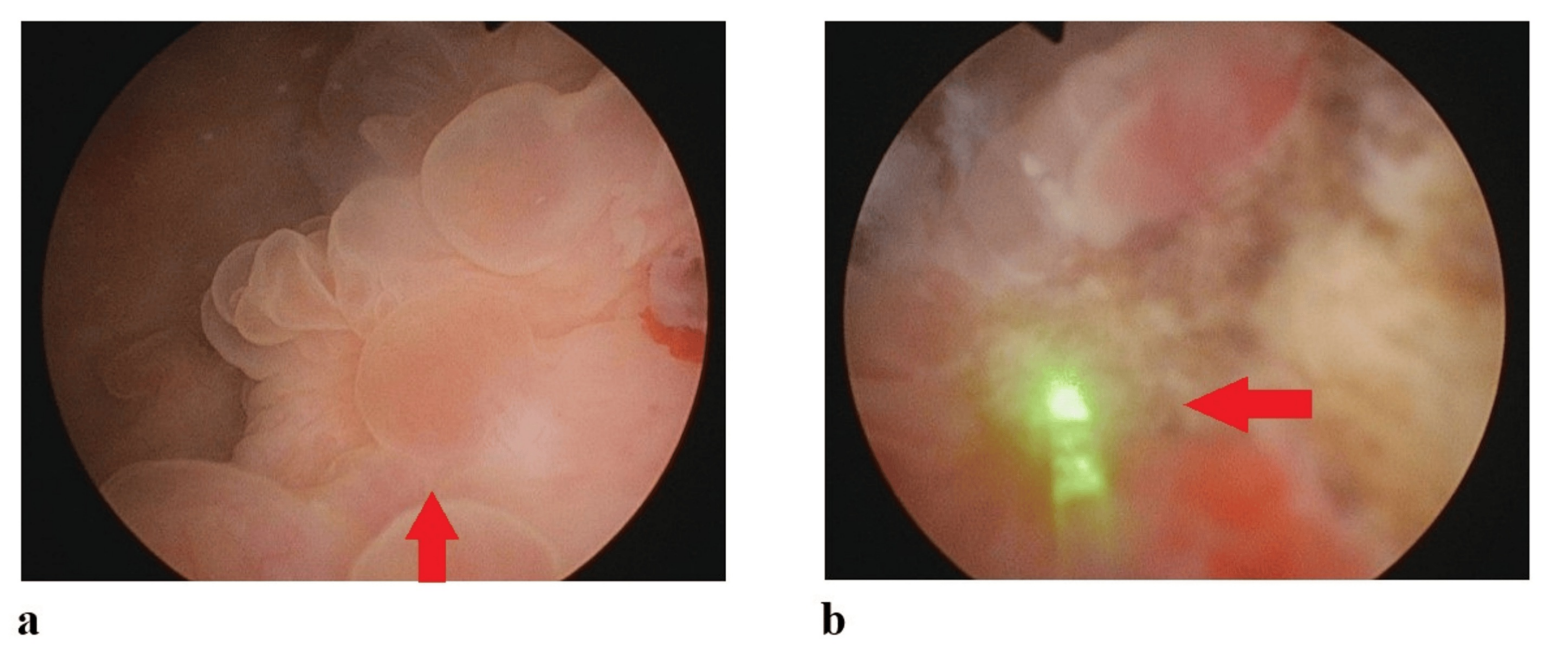
Endoscopic images inside the bladder. (a) Endoscopic image of the bladder with an arrow pointing to the tumor; (b) Endoscopic image with an arrow indicating laser ablation of the bladder tumor.

Initial ultrasound (Figure 1) and CT of the Kidneys, Ureters, and Bladder (KUB) revealed left hydronephrosis and focal bladder wall thickening at the left vesicoureteral junction (VUJ). Subsequent CT with IV contrast depicted a 5.6 × 2.3 cm mass at the distal left ureter/VUJ region, demonstrating peripheral ring enhancement and central hypodensity (Figure 2). MRI further characterized the lesion as an enhancing, partially exophytic mass with possible extravesical involvement and loss of surrounding fat planes (Figure 3). Cystoscopy revealed a large mass involving the bladder trigone and left bladder wall (Figure 4a), obstructing the left ureteric orifice. Laser biopsy and ureteric stent insertion were performed (Figure 4b). Histopathology initially suggested a cauterized papillary urothelial neoplasm of low malignant potential. On follow-up cystoscopy, a more extensive transurethral resection of bladder tumor (TURBT) was undertaken. Histology of resected tissues indicated a papillary urothelial neoplasm of low malignant potential and chronic cystitis. Following a multidisciplinary team (MDT) meeting, further diagnostic and therapeutic cystoscopies and resections were conducted. At the three-month interval, a final cystoscopy involved comprehensive removal of necrotic tissue and deeper resection of the lesion.

Final diagnosis and histopathology

**Figure 5:**
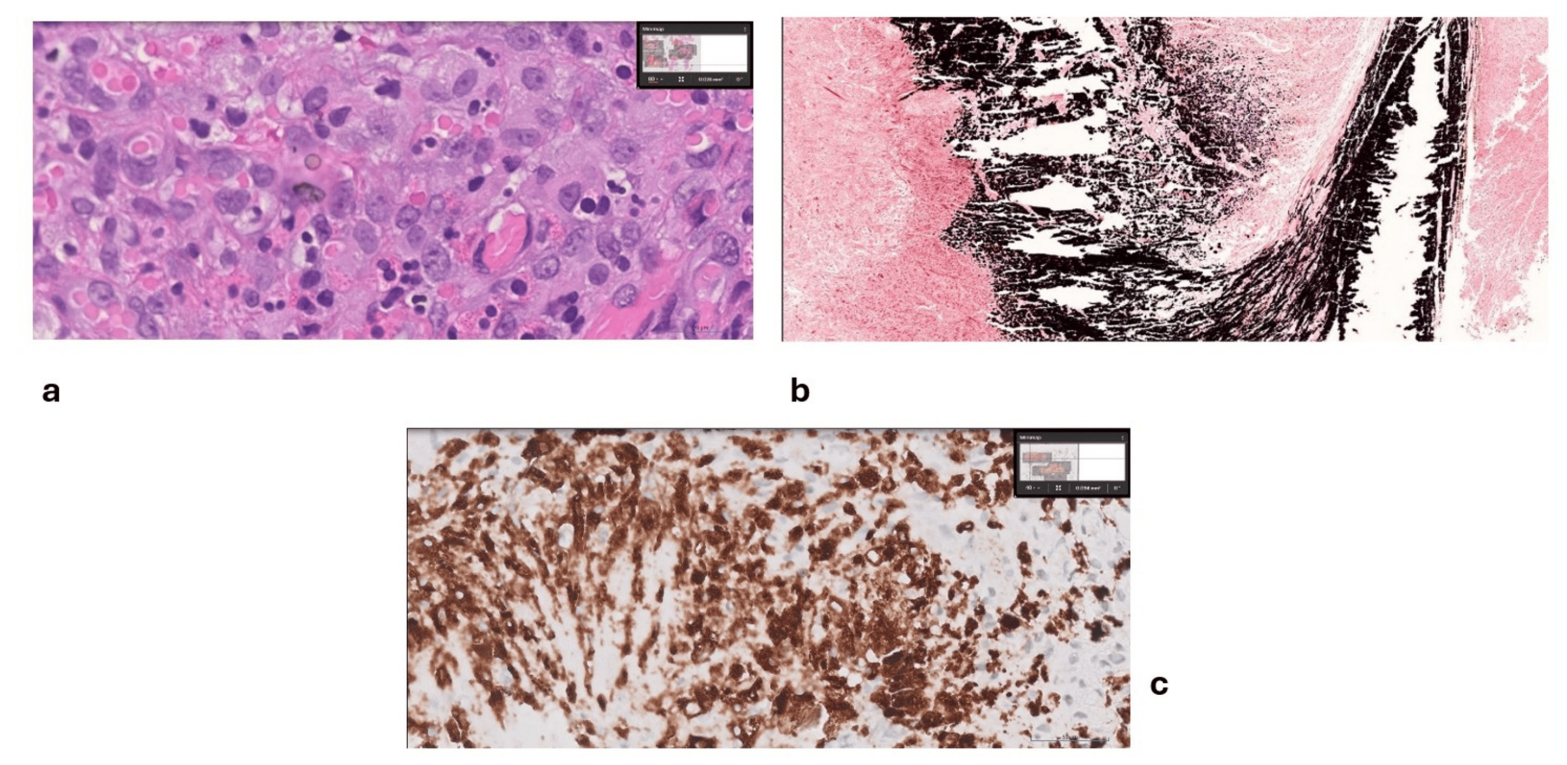
Histopathology slides captured using the Philips IntelliSite Ultra-Fast Scanner. (a) H&E stain at 80x magnification demonstrating abundant macrophages admixed with lymphocytes and eosinophils; (b) Von Kossa stain at 4x magnification showing numerous black calcium deposits, consistent with Malakoplakia; (c) Immunohistochemistry using the CD68 DAKO marker showing positive staining in histiocytes at 40x magnification.

Histopathological analysis from the final procedure indicated a chronic inflammatory disorder characterized by necrosis, calcifications, and granulomatous reactions consistent with malakoplakia (Figure 5a). Special stains such as Von Kossa (Figure 5b), Acid-Fast Bacilli (AFB), and extensive immunohistochemical profiling (CD68, CKAE1/AE3, CD163) were negative for malignancy (Figure 5c), confirming the diagnosis of malakoplakia.

Follow-up and clinical progress

At follow-up, the patient received a 4-week course of fluoroquinolones to complete treatment. The patient reported improvement following the removal of the urethral catheter and stent, with only mild left flank pain persisting. A follow-up CT after 3 months demonstrated resolution of the lesion.

## Discussion

Malakoplakia predominantly affects middle-aged adults, with female patients more commonly presenting with urinary tract involvement. The hallmark nonspecific clinical features, including fever, hematuria, dysuria, and abdominal discomfort, may progress in severe cases to hydroureteronephrosis, documented in approximately 44% of urinary bladder cases [11,12]. Pathogenetically, malakoplakia arises from defective macrophage bactericidal activity, leading to the intracellular persistence of partially digested bacteria and subsequent formation of Michaelis-Gutmann bodies, whose presence is critical for definitive diagnosis [13,2]. *Escherichia coli* remains the most frequently isolated pathogen, although *Klebsiella* spp. and *Proteus* spp. have also been implicated [8,7].

The atypical presentation in the discussed case, an 18-year-old male patient, contrasts with the established epidemiological pattern, underscoring the diagnostic challenges posed by malakoplakia in uncommon demographic groups. Presenting symptoms such as terminal dysuria and gross hematuria, alongside significant imaging findings including hydronephrosis and a mass at the left VUJ, emphasize malakoplakia’s potential to mimic urothelial malignancies, complicating differential diagnosis [14,10]. The patient’s additional systemic symptoms, such as fever and leukopenia despite negative urine cultures, illustrate the diagnostic complexity, as traditional infectious markers and cultures may remain unremarkable in malakoplakia [12].

Histopathological evaluation remains central to definitive diagnosis, with identification of von Hansemann histiocytes and Michaelis-Gutmann bodies facilitated by special staining techniques including periodic acid-Schiff (PAS), von Kossa, and Perl’s Prussian blue stains [1,15]. Immunohistochemical markers, particularly CD68 and CD163, are pivotal in confirming the histiocytic nature of the infiltrates [7,11].

Therapeutically, management strategies emphasize prolonged antibiotic administration, particularly fluoroquinolones and trimethoprim/sulfamethoxazole, due to their superior intracellular penetration capabilities [11]. Surgical intervention, including transurethral resection or nephrectomy, is reserved for obstructive or refractory cases, underscoring the importance of multidisciplinary collaboration in comprehensive patient management [7,13].

Prognostically, early and accurate diagnosis remains crucial, significantly influencing outcomes. Delay in diagnosis or misdiagnosis increases the risk of severe complications, most notably renal impairment and failure [12,8]. This case report highlights the necessity of a multidisciplinary approach, integrating clinical vigilance, precise histopathological identification, and thorough patient monitoring to optimize outcomes. The observed clinical improvement post-intervention and lesion regression on follow-up imaging affirm the efficacy of timely, targeted therapeutic strategies, reinforcing the importance of early intervention and ongoing interdisciplinary cooperation in managing malakoplakia [7,9].

This case broadens clinical understanding of malakoplakia, highlighting its potential for diverse presentations, particularly in younger populations traditionally considered atypical for this disease entity. Increased clinical awareness is essential to facilitate prompt recognition, effective intervention, and optimal patient outcomes in this challenging diagnostic and therapeutic landscape.

## Conclusions

The case highlights a rare presentation of malakoplakia initially mimicking a malignant lesion in the bladder. Multidisciplinary evaluation, detailed histopathological review, and immunohistochemistry were essential in establishing the correct diagnosis. Close clinical and radiological follow-up is advised for early detection of recurrence or complications.
